# Engineered adipose-derived stem cells with IGF-1-modified mRNA ameliorates osteoarthritis development

**DOI:** 10.1186/s13287-021-02695-x

**Published:** 2022-01-15

**Authors:** Haoyu Wu, Zhi Peng, Ying Xu, Zixuan Sheng, Yanshan Liu, Youguo Liao, Yin Wang, Ya Wen, Junzhi Yi, Chang Xie, Xuri Chen, Jiajie Hu, Bingqian Yan, Huijing Wang, Xudong Yao, Wei Fu, Hongwei Ouyang

**Affiliations:** 1 Dr. Li Dak Sum & Yip Yio Chin Center for Stem Cells and Regenerative Medicine, and Department of Orthopedic Surgery of the Second Affiliated Hospital, Zhejiang University School of Medicine, Hangzhou, 310058 China; 2 Department of Sports Medicine, Zhejiang University School of Medicine, Hangzhou, China; 3 Zhejiang University-University of Edinburgh Institute, Zhejiang University School of Medicine, and Key Laboratory of Tissue Engineering and Regenerative Medicine of Zhejiang Province, Zhejiang University School of Medicine, Hangzhou, China; 4 China Orthopedic Regenerative Medicine Group (CORMed), Hangzhou, China; 5 Institute of Pediatric Translational Medicine, Department of Pediatric Cardiothoracic Surgery, Shanghai Children’s Medical Center, School of Medicine, Shanghai Jiao Tong University, Shanghai, 310003 China

**Keywords:** modRNA, IGF-1, Adipose derived stem cells, Gene delivery, Osteoarthritis

## Abstract

**Background:**

Osteoarthritis (OA), a prevalent degenerative disease characterized by degradation of extracellular matrix (ECM), still lacks effective disease-modifying therapy. Mesenchymal stem cells (MSCs) transplantation has been regarded as the most promising approach for OA treatment while engrafting cells alone might not be adequate for effective regeneration. Genetic modification has been used to optimize MSC-based therapy; however, there are still significant limitations that prevent the clinical translation of this therapy including low efficacy and safety concerns. Recently, chemically modified mRNA (modRNA) represents a promising alternative for the gene-enhanced MSC therapy. In this regard, we hypothesized that adipose derived stem cells (ADSCs) engineered with modRNA encoding insulin-like growth factor 1 (IGF-1) were superior to native ADSCs on ameliorating OA development.

**Methods:**

Mouse ADSCs were acquired from adipose tissue and transfected with modRNAs. First, the kinetics and efficacy of modRNA-mediated gene transfer in mouse ADSCs were analyzed in vitro. Next, we applied an indirect co-culture system to analyze the pro-anabolic potential of IGF-1 modRNA engineered ADSCs (named as IGF-1-ADSCs) on chondrocytes. Finally, we evaluated the cell retention and chondroprotective effect of IGF-1-ADSCs in vivo using fluorescent labeling, histology and immunohistochemistry.

**Results:**

modRNA transfected mouse ADSCs with high efficiency (85 ± 5%) and the IGF-1 modRNA-transfected ADSCs facilitated burst-like production of bio-functional IGF-1 protein. In vitro, IGF-1-ADSCs induced increased anabolic markers expression of chondrocytes in inflammation environment compared to untreated ADSCs. In a murine OA model, histological and immunohistochemical analysis of knee joints harvested at 4 weeks and 8 weeks after OA induction suggested IGF-1-ADSCs had superior therapeutic effect over native ADSCs demonstrated by lower histological OARSI score and decreased loss of cartilage ECM.

**Conclusions:**

These findings collectively supported the therapeutic potential of IGF-1-ADSCs for clinical OA management and cartilage repair.

**Supplementary Information:**

The online version contains supplementary material available at 10.1186/s13287-021-02695-x.

## Introduction

Osteoarthritis (OA) is a highly prevalent clinical problem characterized by destruction of articular cartilage, sub-chondral bone sclerosis and synovial inflammation [1]. Current OA pharmacologic therapy including anti-inflammation medication and analgesics, but none of these effectively alter the pathobiological progression of the disease [2]. Recently, mesenchymal stem cells (MSCs) transplantation is regarded as a potential strategy for tissue regeneration attributed to their multi-potency and immunoregulation capability [3, 4]. Series clinical trials have provided evidence that MSCs have clinical utility in modulating OA [5–7]. Among which, adipose derived stem cells (ADSCs) have become indispensable source of stem cell in the field of tissue engineering due to various advantages: (1) evasion of immunogenic and ethical concerns; (2) sustainable source of sample collection amounts with simple, repeatable and minimally invasive methods; (3) great amount and quality of MSCs in adipose tissue [8].

Although results from several preclinical animal and clinical studies are promising, the full potential of ADSCs in tissue regeneration has not yet been realized [9]. Current innovations in genetic engineering techniques have offered vital tools to optimize the application of stem cells for tissue repair purposes [10]. MSCs were engineered to overexpress therapeutic candidate sequences such as insulin growth factor-1(IGF-1) [11], bone morphogenetic proteins (BMPs) [12], and SRY-related HMG-box genes (SOX) family [13], which have shown the great potential to direct stem cells’ chondrogenesis differentiation or promote activation of the anabolic and proliferative processes in damaged cartilage. However, previous gene-enhanced MSC therapy has largely focused on viral DNA-based genetic approaches which are suffered from safety concerns such as poor control of duration, risk of associated tumorigenesis and genomic integration [14]. Messenger RNA (mRNA) transfection represents a safe and effective alternative to DNA-based gene therapy, while its clinical translation has so far been limited by poor stability and strong immunogenicity [15, 16].

Chemically modified mRNA (modRNA) emerged as an attractive approach to express target proteins in vitro as well as in vivo model systems [17]. The stability and protein expression of mRNA transcripts could be improved by modification such as the replacement of modified nucleotides and the addition of a poly(A) tail [18–21]. Recently, Yu et al. applied VEGF modRNA transfected ADSCs to enhance fat survival in a fat graft transplantation model. In this study, the transfection of VEGF modRNA significantly improve the therapeutic efficacy of ADSCs demonstrated by improved neo-angiogenesis, increased graft survival and cell differentiation ability [22]. Together, it suggests that modRNA represents a promising alternative for the gene-enhanced MSC therapy.

modRNA has been broadly advocated on treating bone defect [23], myocardial infarction [14] and critical limb ischemia [18]. However, to our knowledge, the application of modRNA technique in the treatment of OA is extremely limited. Here, we chose to use IGF-1 as our therapeutic sequences. IGF-1 is a growth factor with pleiotropic functions that modulates MSCs chondrogenesis by stimulating proliferation and promoting the chondrogenic differentiation capacity of MSCs [24]. In addition, IGF-1 also plays important roles in cartilage repair that can promote chondrocyte survival, proliferation, and production of cartilage matrix [25]. Due to these characteristics, IGF-1 has garnered considerable interest for OA therapy and showed great potential for clinical translation [26–28]. Herein, we transfected ADSCs with IGF-1 modRNA, characterized the expression pattern of IGF-1-ADSC and explored its potential therapeutic effect in a murine OA model.

## Materials and methods

### Isolation and culture of adipose derived stem cells

ADSCs were isolated as described previously [29]. In brief, Adipose tissues from inguinal fat pad of 6–8-week-old C57BL/6 mice were collected (*n* = 6). After washing with phosphate-buffered saline (PBS), the adipose tissues were then cut into pieces. 0.2% (w/v) Type I collagenase (Invirogen, USA) was used to digest the tissue pieces at 37 °C for 50 min. Culture medium consists of Dulbecco’s modified Eagle’s medium (DMEM, Gibco, USA) supplemented with 10% (v/v) fetal bovine serum (FBS, Gibco, USA) and 1% (v/v) penicillin–streptomycin (PS, Gibco, USA) was used to stop the digestion. Cells were resuspended by growth medium after centrifugation and then seeded onto 10-cm culture dish at 3000 cells/cm^2^. Media were changed every 3 days. ADSCs of the third passage (P3) were used in this study.

### modRNA synthesis and formulation

modRNA was synthesized and formulated as previously described [19]. T7 RNA polymerase-mediated transcription from a linearized DNA template, which incorporates generic 5′and 3′UTRs and a poly-A tail, was used for mRNA synthesis. The purification of RNA was performed using Ambion MEGA clear spin columns. RNA was then treated with Antarctic Phosphatase (New England Biolabs) at 37 °C for 30 min to remove residual 5′-phosphates. After re-purification and quantification by Nanodrop (Thermo Scientific), RNA was resuspended in 10 mM Tris HCl, 1 mM EDTA at 1 μg/μl for use. In mRNA, uridine was fully replaced by N1-methylpseudouridine. GFP and firefly luciferase ORF sequences were the same as previously described [14]. Open reading frame sequence for mouse IGF-1 modRNA was provided in the supplementary data (Additional file 1: Table S1).

### modRNA transfection in vitro

MessengerMAX (Invitrogen, USA) transfection reagents was used to carry out modRNA transfections. Transfection reagents and modRNA were respectively diluted in Opti-MEM media (Invitrogen, USA) and then incubated for 5 min. Subsequently, the mixes were pooled together and incubated at room temperature (RT) for 15 min to generate the transfection mixture. 5 μl of MessengerMAX transfection reagent were applied per 2 μg of RNA. Cells were incubated with the transfection mixture for 4 h. 2 μg RNA was applied for transfection of 1.5 × 10^5^ ADSCs in accordance with our previous study [18, 22].

To evaluate GFP modRNA expression, the transfected ADSCs were photographed at 24 h after transfection. Fluorescence intensity and transfection efficiency of GFP-modRNA was analyzed by confocal microscopy and flow cytometry at 24 h after transfection. Cells were then harvested using trypsin (0.05% (w/v); Gibco, Cat. 15400054), washed with PBS completely and resuspended in 500 μl PBS. Samples were analyzed on a FC500MPL flow cytometer (Beckman Coulter, USA). Data of flow cytometry were analyzed by FlowJo vX.0.7 software.

To measure the protein expression kinetics of IGF-1 modRNA, the supernatant of IGF-1 modRNA transfected ADSCs was harvested at specific time point (4, 16, 24, 48, 72, 96, 120 h post transfection) and then the concentration of IGF-1 protein was quantified by ELISA (MultiSciences, China). To collect the conditioned medium of transfected ADSCs, medium was then replaced by culture medium (DMEM supplemented with 10% FBS).

### Isolation and culture of chondrocytes

Mouse chondrocytes were isolated according to previous studies [30, 31]. Articular cartilage tissue of postnatal day 5–6 C57BL/6 mice (*n* = 6) was collected. The cartilage tissue was washed in PBS and cut into pieces. Type II collagenase (Invitrogen, USA) was used to digest the tissue pieces at 37 °C overnight. Culture medium containing DMEM/F12 (Gibco, USA) with 10% (v/v) FBS and 1% (v/v) PS was used to stop the digestion. Cells were resuspended by culture medium after centrifugation and then plated on 10-cm culture dish at 37 °C, 5% CO_2_ environment. Chondrocytes of the second passage (P2) were used in this study.

### Chondrocytes treated by recombinant and purified IGF-1 modRNA produced IGF-1 protein

1.5 × 10^5^ mouse chondrocytes were seeded into 6-well plates. Cells were starved in F12/DMEM for 24 h and then exposed to 20 ng/ml IGF-1, either recombinant protein (Peprotech, USA) or protein produced from IGF-1 modRNA (added as conditioned media), or to control media. Stimulation was carried out for 48 h, and total protein of chondrocytes was extracted using RIPA lysis buffer comprising of protease and phosphatase inhibitors (Solaribio, China).

### Western blot analysis

The BCA Protein Assay kit (Pierce, USA) was applied to measure the concentration of extracted protein. Protein was then separated on SDS–polyacrylamide gel electrophoresis gels by electrophoresis. Then protein was transferred onto a polyvinylidene difluoride membrane. Membrane was blocked in 1% (w/v) bovine serum albumin (BSA) at RT for 1 h and subsequently incubated with primary antibodies of mouse Col2 (Santa Cruz, sc-52658, dilution: 1:200) at 4 °C overnight. Membrane was washed in Tris-buffered saline with Tween (TBST) and incubated with horseradish peroxidase (HRP) secondary antibodies (goat anti-mouse, Beyotime Institute of Biotechnology, China, A0216, dilution:1:1000) at RT for 1.5 h. After washed by TBST to rinse off the excess antibody, membrane was then subjected to western blot detection reagents (ECL, Thermo Scientific, USA) to generate chemiluminescent signal based on manufacturer's protocol.

### Cell coculture

Non-contacting coculture system (transwell chambers (Corning, USA) with a 0.4-μm pore polycarbonate membrane insert) was applied to investigate the effect of IGF-1-ADSCs on mouse chondrocytes. Chondrocytes without coculture were used as control. ADSCs transfected with GFP modRNA were used as a transfection control (GFP-ADSCs) in the experiments. ADSCs, GFP-ADSCs or IGF-1-ADSCs were respectively plated onto the membrane of transwells, and chondrocytes were plated onto the bottom of 24-well plates. Human interleukin 1 beta (IL-1β) (Peprotech, USA) was added to the medium (10 ng/ml) [32]. These treated chondrocytes were collected for RNA preparation and immunofluorescence at 48 h after treatment.

### qPCR analysis

The mRNA was extracted using Trizol (Invitrogen, USA) followed by reverse transcription. PCR was performed using the SYBR Green Real-time PCR Master Mix kit (Takara, Japan) based on the manufacturer’s instructions with a Light Cycler apparatus (ABI 7900HT). The following primer sequences were used: Col2a1 sense 5'- CCACA CCAAA TTCCT GTTCA-3', antisense 5'-ACTGG TAAGT GGGGC AAGAC-3'; Acan sense 5'-CCACA CCAAA TTCCT GTTCA-3', antisense 5'-ACTGG TAAGT GGGGC AAGAC-3'; Igf-1 sense 5'-CACAT CATGT CGTCT TCACA CC-3', antisense 5'-GGAAG CAACA CTCAT CCACA ATG-3'; Gapdh sense 5'-ATACG GCTAC AGCAA CAGGG-3', antisense 5'-TGTGA GGGAG ATGCT CAGTG-3'. Relative level of expression of target genes was then calculated using the 2-ΔΔCt method and the representative results are presented as target gene expression normalized to the reference gene Gapdh.

### Immunofluorescence

The control ADSCs, GFP-ADSCs and IGF-1-ADSCs were harvested 16 h after transfection. The expression of IGF-1 (Abcam, ab106836, dilution: 1:50) was detected. The chondrocytes were harvested 48 h after treatment. Immunofluorescence was used to evaluate the anabolic markers of the chondrocytes. The anabolic markers including Col2(Santa Cruz, sc-52658, dilution: 1:200) and Aggrecan (Abcam, ab36861, dilution: 1:200) were detected. Cells were firstly fixed in 4% (vol/vol) paraformaldehyde for 30 min and subjected to immunofluorescence for primary anti-bodies at 4 °C overnight. After washed by PBS, cells were incubated with secondary anti-bodies (Alexa Fluor® 488, A21202 for Col2, dilution: 1:500; Alexa Fluor® 546, A11035 for Aggrecan, dilution: 1:500; Cy3, Beyotime Institute of Biotechnology, A0502 for IGF-1, dilution: 1:500) and the nucleus was visualized with DAPI. The sections were viewed under a Nikon A1R confocal laser scanning microscope (Nikon, Tokyo, Japan).

### Bioluminescence imaging

Animal experimental protocols were approved by Zhejiang University Ethics Committee (ZJU16059). In vivo imaging system (IVIS, Caliper Life Sciences, USA) was used to demonstrate the bioluminescence imaging of transplanted ADSCs which are transfected with modRNA encoding luciferase (Luc modRNA). Male 10-week-old C57BL/6 mice were randomly divided into 2 groups (*n* = 3 per group). 2 × 10^5^ ADSCs (ADSCs group) or Luc-ADSCs (Luc modRNA group) were respectively injected into the right knee joints of mice with a dose of 1 μg RNA/7.5 × 10^4^ ADSCs. At 16 h, 24 h, 48 h, 72 h after treatment, mice were imaged. Mice received intra-articular injection of d-luciferin (Solarbio, China) at the dose of 150 μg/10 μl. After 5 min, mice were anesthetized by isoflurane inhalation. The exposure time was set at 10 s and the values of bioluminescence were quantified by measurement of photon flux.

### In vivo cell tracking

Male 10-week-old C57BL/6 mice were randomly divided into 3 groups (*n* = 3 per group). 2 × 10^5^ ADSCs, GFP-ADSCs or IGF-1-ADSCs were staining with DiI (invitrogen, USA) and respectively injected into the knee joints of mice. To evaluate the cell viability in knee joint, in vivo imaging system (IVIS, Caliper Life Sciences, USA) was used to demonstrate the fluorescence signal at 1 week and 4 weeks after transplantation.

### OA animal model induced

Surgical destabilization of medial meniscus (DMM) was performed on the right hind knees of Male 10-week-old C57BL/6 mice (*n* = 40) according to previous study [33], with Sham surgery performed on the contralateral left knees. Briefly, the knee articular cavity was opened after anesthesia and the medial meniscus was destabilized by cutting medial meniscotibial ligament. The Sham surgery was performed by opening the joint capsule without further damage. At 1 week and 2 weeks after surgery, the OA induced knee joint were equally divided to four groups randomly and intra-articular injections of DMEM (Control), ADSCs, GFP-ADSCs or IGF-1-ADSCs (2 × 10^5^ cells in 10 μl DMEM [34, 35]) were performed respectively with a 0.5-ml monoject (29-gauge) insulin syringe (BD, USA). After surgery, animals were allowed free cage activity. Terramycin (60 mg/kg) will be administered subcutaneously as antibiotic prophylactic at every 72 h basis. Flunixin (by adding 0.1 ml of 50 mg/ml flunixin to 200 ml water) will be used for 3 days after surgery as analgesic. Mice were housed in cage after the surgery and running wheels were put in 2 days after the surgery to encourage exercise. The mice were harvested at 4 weeks and 8 weeks after surgery and the knee joints were collected for histological evaluation. Each experimental group included a total of 5 samples.

### Tissue fixation and histological analysis

At 4 weeks and 8 weeks after surgery, the harvested knee joints were firstly fixed in 4% (vol/vol) paraformaldehyde for 24 h and then decalcified in 10% (wt/vol) ethylene diamine tetra-acetic acid (EDTA) solution for 1 month. Knee joints were embedded in paraffin blocks, slice (7 μm), stained with Safranin Orange (SO) and OARSI scored [36] sequentially in a blinded manner by 3 observers (YX, ZXS and YGL) (3 sections were scored per knee).

### Immunohistostaining

Paraffin sections were firstly treated with 0.5% pepsin (Sangon Biotech, China) at 37 °C for 30 min. Subsequently treated with 3% hydrogen peroxide in methanol for 10 min to block endogenous peroxidase and then with 5% BSA to block nonspecific protein binding. After incubated with primary antibody (mouse anti-Col2 (Santa Cruz, USA, sc-52658, dilution: 1:200) and mouse anti-Aggrecan (Abcam, USA, ab36861, dilution: 1:200)) at 4 °C overnight (2% BSA was used as negative control), sections were incubated with secondary antibodies [goat anti-mouse (Beyotime Institute of Biotechnology, China, A0216, dilution:1:50) or goat anti-rabbit (Beyotime Institute of Biotechnology, China, A0208, dilution:1:50)] at RT for 2 h. The sections were then washed in distilled water for 5 min and incubated with DAB (Beyotime Institute of Biotechnology, China, P0202) at RT for 3 min. The stained specimens were photographed digitally under a slide scanning machine (Pannoramic MIDI, 3DHISTECH Ltd., Budapest, Hungary).

### Statistical analysis

All data were shown as the mean ± SE. Statistical analysis was performed and charts were constructed with GraphPad Prism version 8.0. Statistical significance (*P* < 0.05) was calculated using unpaired two-tailed Student’s *t*-tests (two groups at the same time point), one-way ANOVA (multiple groups at the same time point). Statistical results of the in vivo experiment were analyzed by the Mann–Whitney test using the SPSS software 18.0.

## Results

### Efficient transfection of chemically modified mRNA in mouse ADSCs in vitro

**Fig. 1:**
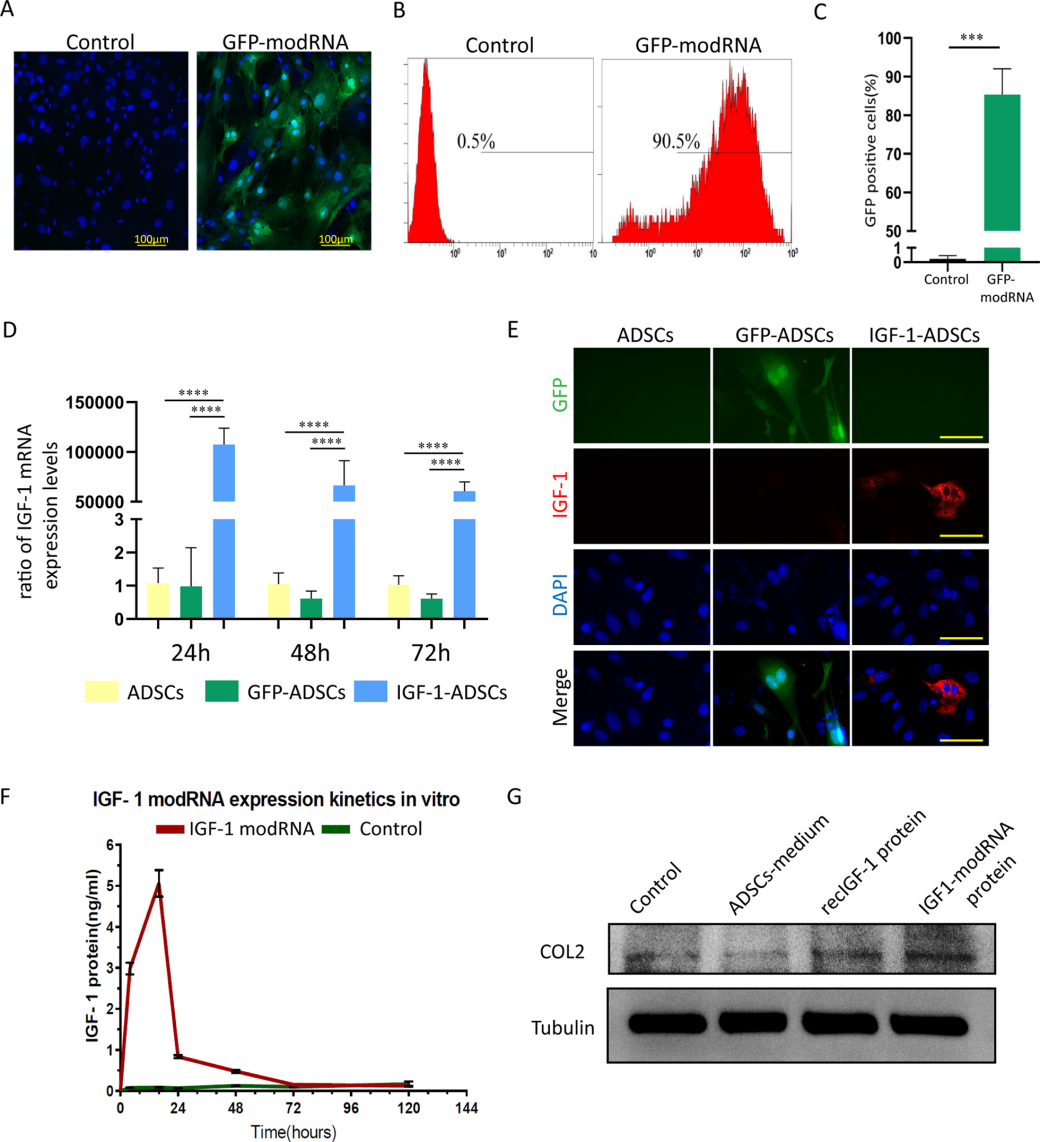
Efficiency and protein kinetics of modRNA transfected ADSCs. **A** Representative images demonstrating GFP expression in GFP modRNA transfected ADSCs (the GFP-modRNA group) at 24 h after transfection (Scale bar = 100 μm) compared to ADSCs (the control group). **B**, **C** Transfection efficiency by flow cytometry analysis at 24 h after transfection. **D** Expression levels of IGF-1 mRNA in ADSCs, GFP-ADSCs and IGF-1-ADSCs at 24 h, 48 h and 72 h post-transfection. **E** Representative images demonstrating expression of IGF-1 protein in ADSCs, GFP-ADSCs and IGF-1-ADSCs at 16 h post-transfection. (Scale bar = 60 μm) **F** ELISA analysis of kinetics of IGF-1 protein from IGF-1 modRNA transfected mouse ADSCs (native ADSCs, the control group). **G** Western blot analysis of the type II collagen production of chondrocytes treated with the medium of ADSCs, recombinant IGF-1 protein or IGF-1 protein produced by IGF-1-ADSCs. (*P < 0.05, **P < 0.01, ***P < 0.001, *ns* not significant)

We synthesized modRNA by previous protocol [18, 37]. A GFP reporter was utilized to demonstrate the protein expression kinetics and transfection efficiency of modRNA in mouse ADSCs. The expression level of GFP was measured by flow cytometry after 24 h. Compared to native ADSCs, a clear GFP fluorescence presented in the ADSCs transfected with GFP modRNA (Fig. 1A). Moreover, the results demonstrated that 85 ± 5% of cells expressed GFP, indicating the high transfection efficiency of modRNA (Fig. 1B, C).

In order to determine the expression dynamics of modRNA in ADSCs, we respectively transfected ADSCs with either GFP modRNA or IGF-1 modRNA and detected transcript levels inside the cells. Quantitative reverse-transcription polymerase chain reaction (qRT-PCR) revealed more than 50,000-fold increase of IGF-1 mRNA expression in the IGF-1-ADSCs group at 24 h, 48 h and 72 h after transfection, in comparison to the GFP-ADSCs and ADSCs (Fig. 1D). Using immunofluorescence, we demonstrated that the intracellular levels of IGF-1 protein in the IGF-1-ADSCs group expressed significantly more IGF-1 protein 16 h after transfection than the ADSCs and GFP-ADSCs groups, which further confirmed the translation of the modRNA (Fig. 1E). To test if the IGF-1 modRNA transcript could translate into secreted and functional protein, the expressional protein kinetics of IGF-1 modRNA in ADSCs were tested by enzyme-linked immunosorbent assay (ELISA). As shown in Fig. 1F, IGF-1 production was immediately increased and reached high level after only 4 h, peaked at 16 h, and returned to baseline at 72 h. To confirm the bio-functionality of IGF-1 protein produced from IGF-1 modRNA transfected ADSCs, we introduced the same concentration of IGF-1 modRNA produced protein and commercially available recombinant mouse IGF-1 protein to mouse chondrocytes. After 48 h, the expression of type II collagen of chondrocytes was assessed. Notably, IGF-1 modRNA produced protein exhibited comparable type II collagen production with recombinant IGF-1 protein, while the additional control group from native ADSC supernatant showed much lower type II collagen production (Fig. 1G). These results demonstrated that IGF-1 modRNA transfection could generate high level and bio-functional IGF-1 to boost chondrocytes type II collagen expression.

### ADSCs transfected with IGF-1 modRNA promoted the extracellular matrix synthesis of chondrocytes in vitro

**Fig. 2:**
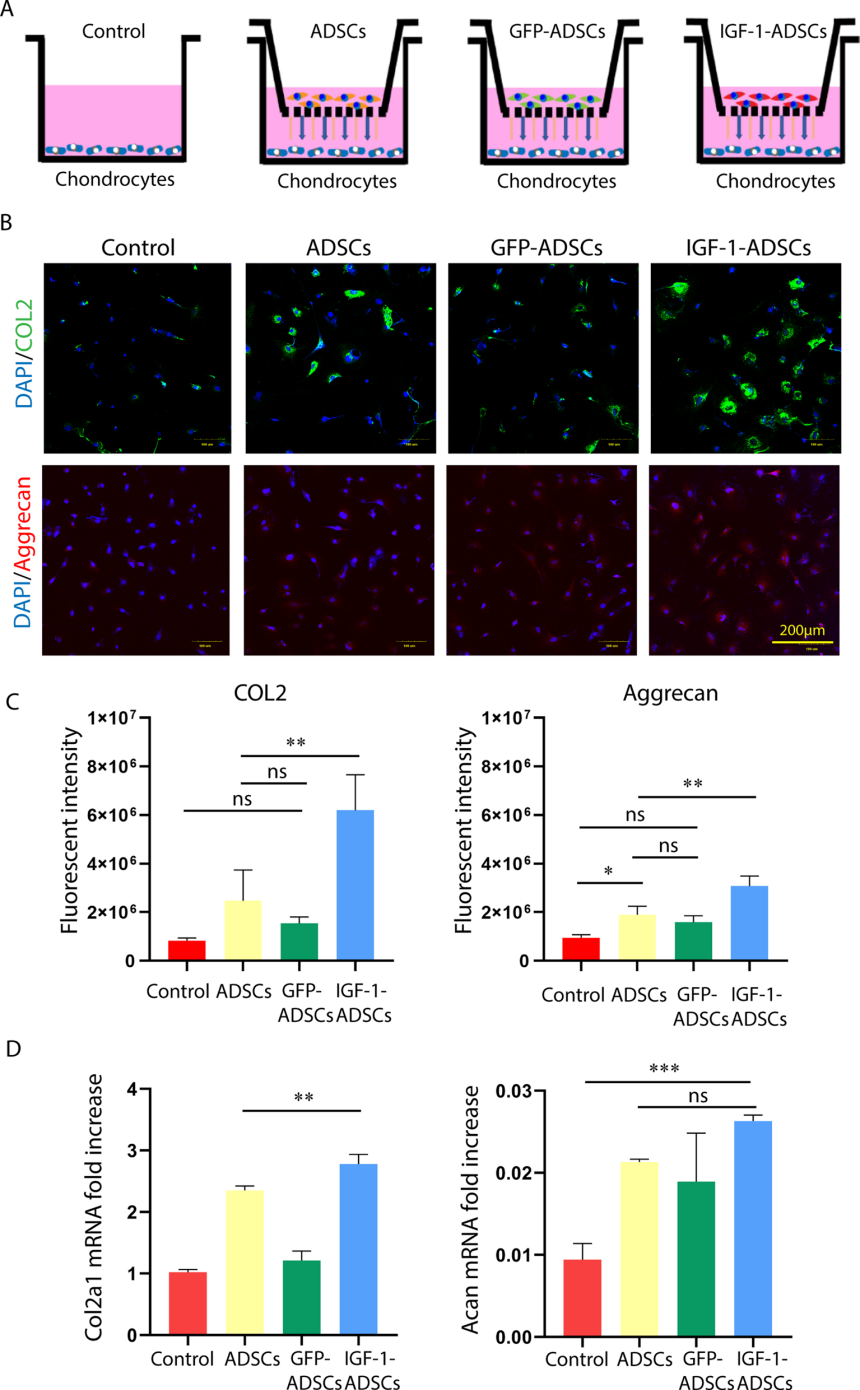
ADSCs transfected with IGF-1 modRNA promoted extracellular matrix synthesis of chondrocytes. **A** Schematic model of indirect co-culture system of chondrocytes with ADSCs, GFP-ADSCs or IGF-1-ADSCs using transwell in the presence of IL-1β (chondrocytes without co-culture, the control group). Each cell type was grown independently on the transwell plates. **B**, **C** Representative photo-micrographs and fluorescent intensity analysis of immunofluorescence staining for aggrecan and type II collagen in mouse primary chondrocytes cocultured with ADSCs, GFP-ADSCs or IGF-1-ADSCs for 48 h (Scale bar = 100 μm). **D** Quantitative polymerase chain reaction analysis of mRNA transcript levels of Col2a1 and Acan in mouse primary chondrocytes cocultured with ADSCs, GFP-ADSCs, IGF-1-ADSCs for 48 h (chondrocytes without co-culture, the control group). (*P < 0.05, **P < 0.01, ***P < 0.001, *ns* not significant)), *COL2* type II collagen, *IL-1β* interleukin 1 beta

We next used a co-culture system to investigate whether IGF-1-ADSCs stimulate the synthesis of cartilage-anabolic proteins (type II collagen and aggrecan) in chondrocytes, as this is a critical factor for cartilage tissue regeneration. To better mimic the OA environment in vitro, interleukin 1 beta (IL-1β) was added to the medium [32]. The ADSCs transfected with GFP modRNA (GFP-ADSCs) were used as a transfection control in the experiments. As shown in Fig. 2A, chondrocytes were respectively co-cultured with ADSCs, GFP-ADSCs or IGF-1-ADSCs in a transwell system (chondrocytes without co-culture were used as control). After 48 h, the expression level of type II collagen and aggrecan in chondrocytes was assessed. The immunofluorescent staining results showed that expression of type II collagen and aggrecan were significantly higher in the IGF-1-ADSCs/chondrocyte co-culture group (*P* < 0.05), when compared with the other three groups (Fig. 2B, C). qRT-PCR results also showed the expression of Col2a1 and Acan genes were significantly higher in chondrocyte co-cultured with IGF-1-ADSCs (Fig. 2D). These results suggested that IGF-1-ADSC significantly increased the anabolic markers of chondrocytes in inflammatory microenvironment.

### Highly efficient protein expression of modRNA-transfected ADSCs in vivo

**Fig. 3:**
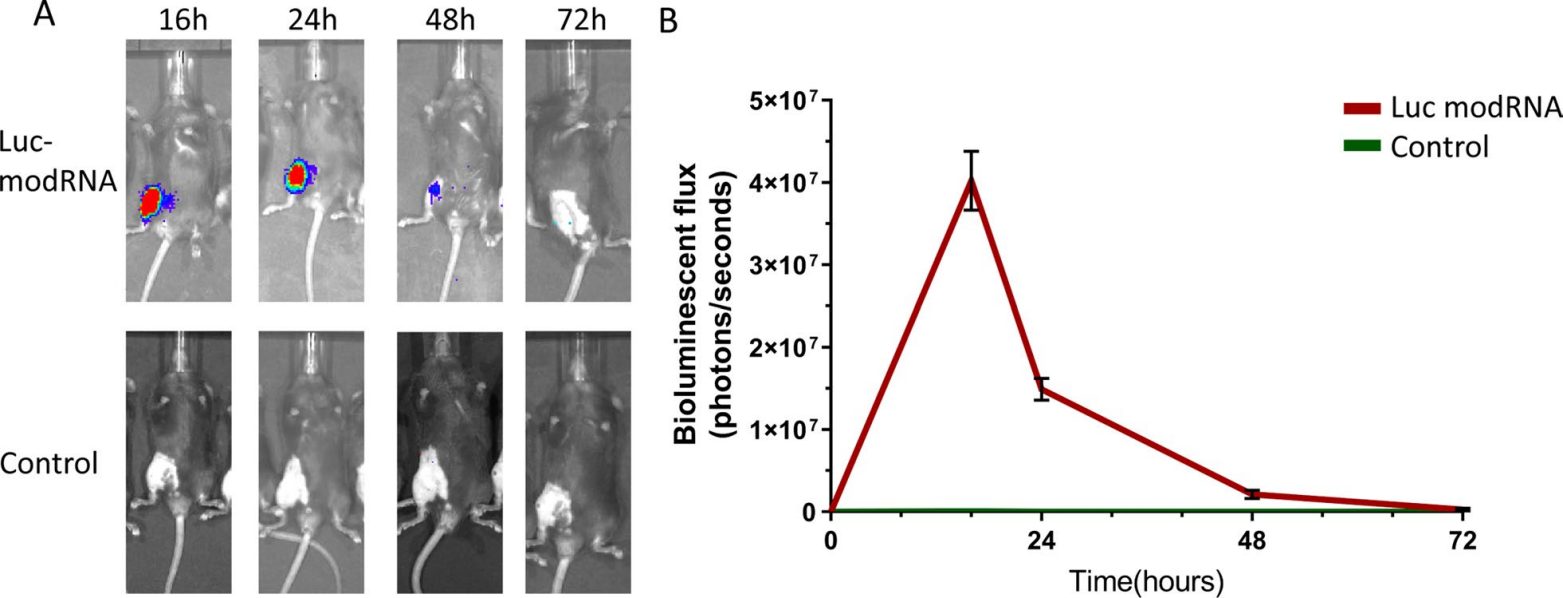
The in vivo expression kinetics of modRNA transfected ADSCs. **A** Representative imaging of mice which received intra-articular transplantation of Luciferase modRNA (Luc modRNA) transfected ADSCs (native ADSCs, the control group). **B** Luciferase activity in the knee joint after transplantation

To determine the expression kinetics of modRNA in vivo, ADSCs were transfected with luciferase-modRNA (Luc modRNA group) and transplanted into mice knee joint. The luciferase signals were detected by IVIS bioluminescence imaging. The results showed that luciferase expression from the Luc modRNA group peaked the highest expression at 16 h and retained for about 72 h, which was in line with the potent and transient burst of gene expression in vitro (Fig. 3A, B).

### Transplantation of IGF-1 modRNA transfected ADSCs attenuated the degeneration of extracellular matrix in mice suffering from OA

**Fig. 4:**
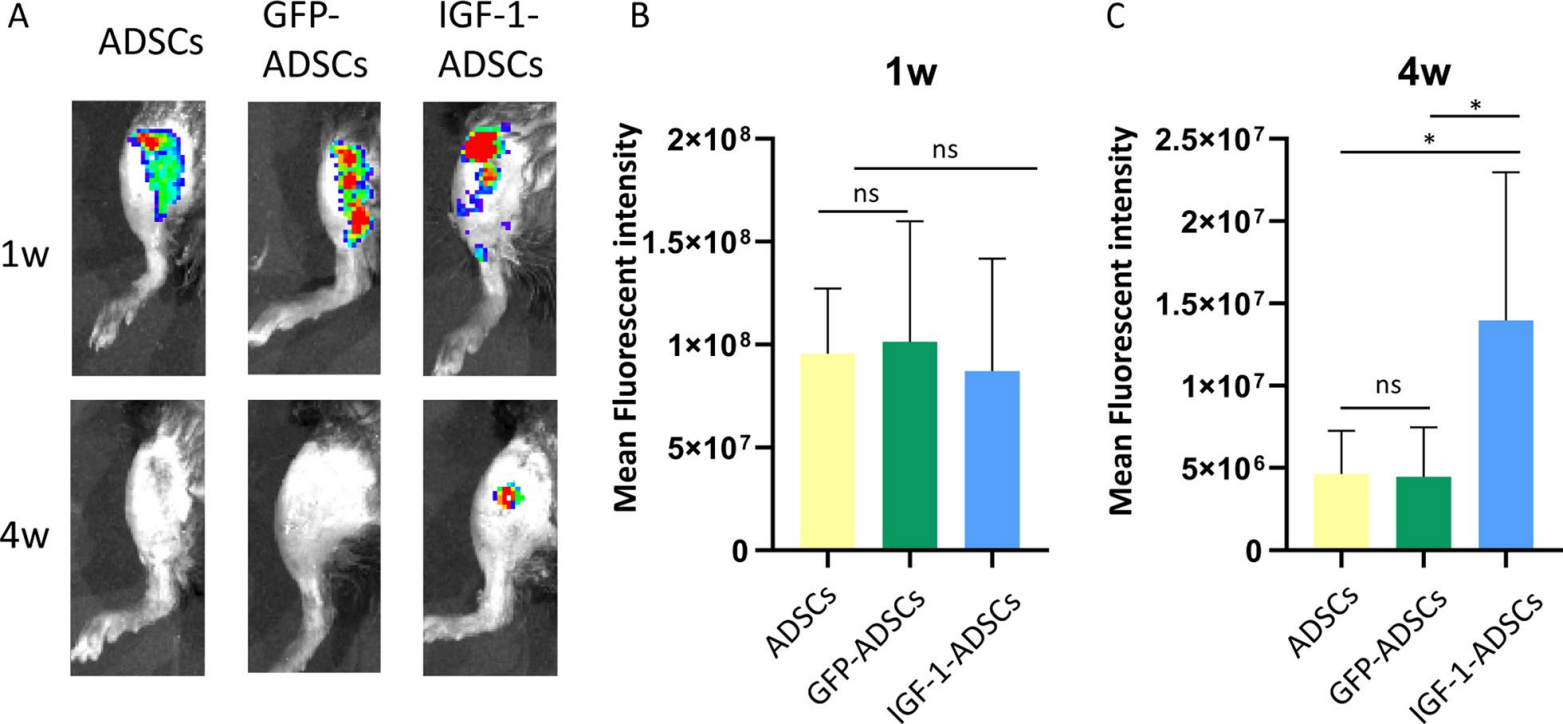
The cell survival of IGF-1 transfected ADSCs after intra-articular transplantation. **A** Tracking cell survival after intra-articular transplantation of ADSCs, GFP-ADSCs or IGF-1-ADSCs using DiI labeling. **B**–**C** Fluorescent signals in the knee joint at 1 week(B) and 4 weeks(C) after intra-articular transplantation (*n* = 3/group; ns = *P* > 0.05, *P < 0.05, **P < 0.01)

Given IGF-1-ADSC could increase the chondrocyte anabolic markers expression under inflammation in vitro, we set out to analyze whether it might attenuate OA progression. Firstly, the survival rate of transplanted cells was examined. ADSCs, GFP-ADSCs, IGF-1-ADSCs were respectively labelled with DiI and transplanted into knee joints. The results showed that the injected cells in the three groups could be tracked after 1 week. However, after 4 weeks, the signal was only detectable in IGF-1-ADSCs groups, which indicated that IGF-1 modRNA transfection could increase the cell viability of transplanted ADSCs in vivo (Fig. 4A–C).

**Fig. 5:**
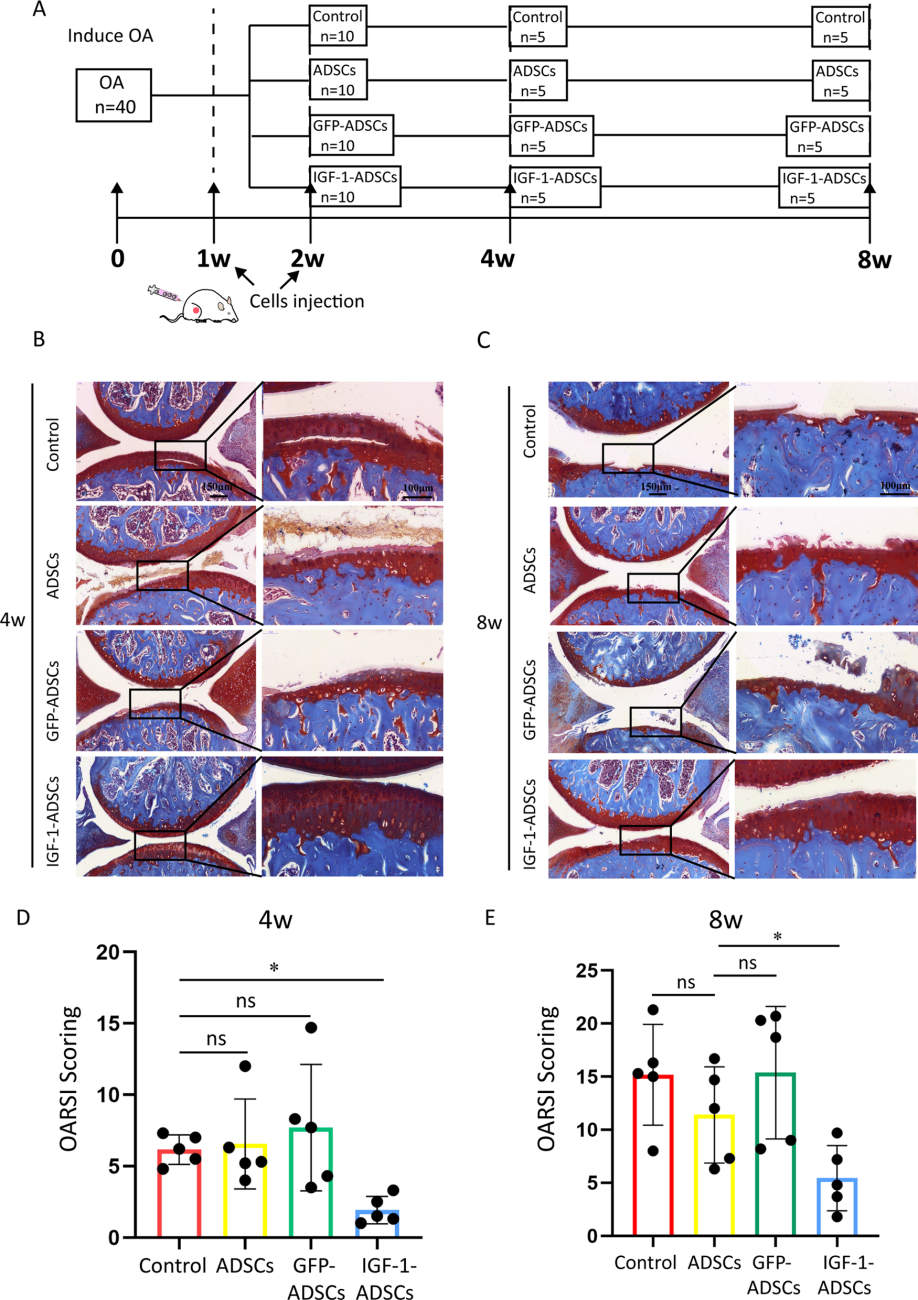
Intra-articular transplantation of IGF-1-ADSCs ameliorated OA cartilage degradation. **A** Schematic illustration of in vivo experiment design: the intra-articular injection was performed twice at 1 week and 2 weeks after surgery. Samples were harvested at 4 weeks or 8 weeks after surgery. **B**, **C** Typical photomicrographs of SO staining of mouse knee joints at 4 weeks (**B**) and 8 weeks (**C**) post-surgery. (Scale bar = 150 μm (left) or 100 μm (right)). **D** OARSI Scoring of mouse knee joints at 4 weeks (**D**) and 8 weeks (**E**) post-surgery. (ns = *P* > 0.05, *P < 0.05, **P < 0.01)

**Fig. 6:**
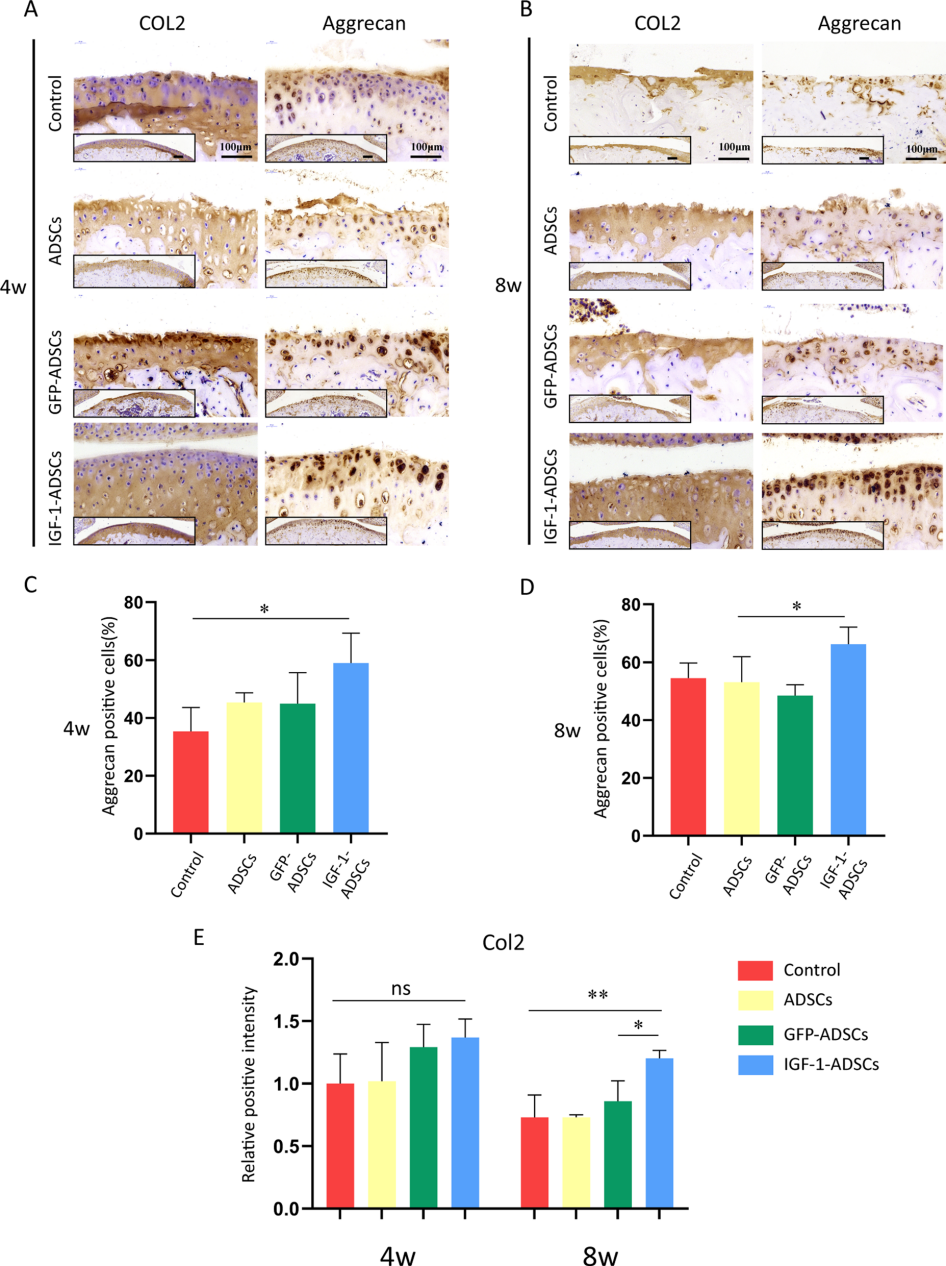
Intra-articular transplantation of IGF-1-ADSCs decreased the loss of extracellular matrix. **A**, **B** Immunohistochemistry staining of mouse knee joints for aggrecan and type II collagen at 4 weeks (**A**) and 8 weeks (**B**) post-surgery (Scale bar = 100 μm). **C**, **D** The ratio of Aggrecan positive cells in cartilage samples at 4 weeks (**C**) and 8 weeks (**D**) after surgery. **E** The relative positive intensity of type II collagen staining in cartilage samples at 4 weeks and 8 weeks after surgery (ns = *P* > 0.05, *P < 0.05, **P < 0.01)

To investigate whether transplantation of IGF-1-ADSCs can delay OA progression, IGF-1-ADSCs were injected intraarticularly into mice OA joints at 1 week and 2 weeks post-surgery and knee joints were collected at 4 weeks and 8 weeks after surgery for further evaluation (Fig. 5A). The result showed that ADSCs and GFP-ADSCs group exhibited similar OA development with the untreated control group, which had a thin cartilage surface and decrease Safranin Orange (SO) staining (Fig. 5B, C). The Osteoarthritis Research Society International (OARSI) score results also showed no significant difference among these three groups (Fig. 5D, E). However, the IGF-1-ADSCs transplantation markedly prevented the articular cartilage degeneration as assessed by increased SO staining and decreased OARSI score. Moreover, expression levels of Aggrecan and Type II collagen were evaluated by immunohistochemistry (Fig. 6A, B). The results demonstrated that IGF-1-ADSCs effectively restored ECM deposition demonstrated by higher expression of Aggrecan and Type II collagen compared to the other groups (Fig. 6C–E). Collectively, these results revealed that the transplantation of IGF-1-ADSCs was effective in delaying OA progression in mice OA model.

## Discussion

**Fig. 7:**
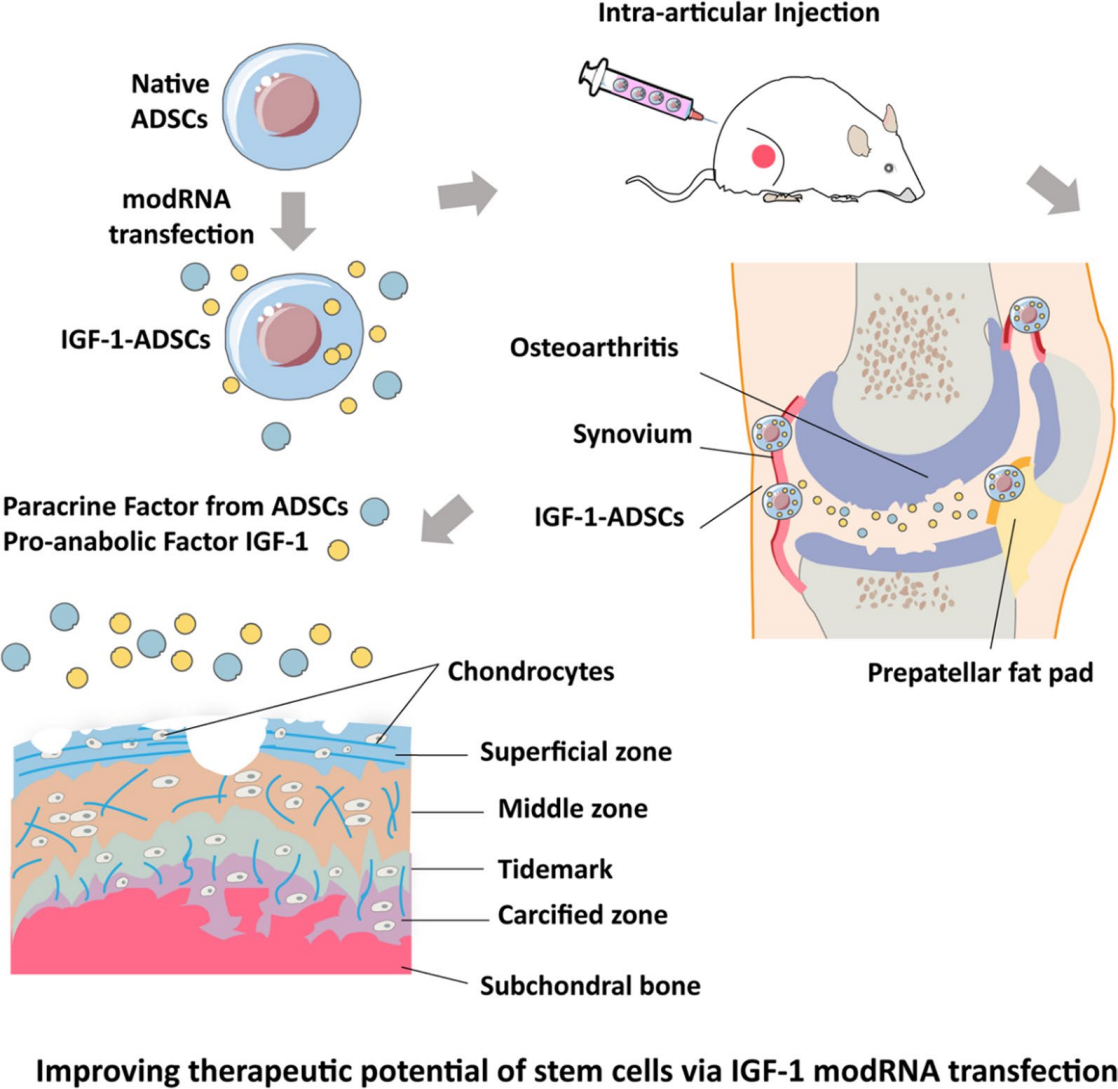
Improving the therapeutic potential of stem cells via IGF-1 modRNA transfection

In this study, we reported the first study emphasizing the therapeutic potential of combining ADSCs with modRNA technologies for OA treatment (Fig. 7). First, we successfully developed genetically modified ADSCs to secrete trophic factor IGF-1 by modRNA technique. Second, our result demonstrated that the presence of IGF-1-ADSCs could significantly increase the expression of anabolic markers of chondrocytes in the inflammatory microenvironment. Third, the result showed that IGF-1-ADSCs displayed superior chondroprotective effect over native ADSCs in murine OA model, highlighting that this therapeutic management might be potential for ameliorating OA progression.

Transplantation of stem cells undoubtedly represents a promising strategy to combat OA [3]. Various populations of MSCs such as ADSCs [3], bone marrow mesenchymal stem Cells (BMSCs) [5, 6], and umbilical cord-derived stem cells (UC-MSCs) [7, 38] have been employed in clinical practice. BMSCs were reported to be the most common MSC population applied in the orthopedic field [39], while ADSCs were recently emerging as alternatives to BMSCs. ADSCs have several key advantages including relatively more abundant sources, evasion of ethical concerns and rapid amplification [40]. In addition, we previously reported that ADSCs were more adaptable to surviving in the hypoxic articular cavity niche and exhibited superiority in modulating inflammation when compared to BMSCs [41]. Collectively, these advantages implicate that ADSCs are ideal stem cell source for OA treatment.

Although ADSCs transplantation displayed great therapeutic potential, several pertinent limitations including low cell survival rate and low efficacy hindered advances in stem cell therapy. Combing with other engineering approaches such as recombinant proteins or DNA vectors appears to be an effective strategy to realize the full potential of ADSCs in regenerative medicine [10]. In this study, we did not investigate the application of IGF-1 recombinant proteins due to their short half-life and poor pharmacokinetic properties [42]. On the other hand, modifying MSCs through DNA-based genetic manipulations raises various safety concerns such as poor control of duration and risk of tumorigenesis which limits its clinical translation [17]. modRNA-based method has the potential to be a more direct and efficient way for the gene-enhanced MSC therapy without the above issues related to DNA or viral vectors [22]. The advantages of modRNA approach, when compared to DNA-based transfection, included high transfection efficiency and the ability of transient and considerable expression of target protein [17]. As shown in Fig. 1, modRNA transfected primary mouse ADSCs with high efficiency (85 ± 5%). This high efficiency represented a significantly increase compared to typical transfection efficiency when applied non-viral DNA mediated transfection [43, 44]. IGF-1, as a naturally secreted substance of ADSCs, has anabolic and chondroprotective properties on the cartilage, while low levels of endogenously secreted IGF-1 are inadequate for effective regeneration [45]. In this study, we generated genetically modified ADSCs with the ability to secrete IGF-1 in a transient and potent manner using modRNA. Our results showed the IGF-1 protein production using modRNA was approximately 10 folds increase compared to the previous study using DNA in 24 h after transfection [46]. This is important when considering the therapeutic efficiency of IGF-1 is dose-dependent [47].

Previous researches reported that the therapeutic potential of MSCs transplantation mainly depends on their paracrine function rather than differentiation potential [48]. A series of studies have proposed “hit-and-run” MSC mode of action upon transplantation [49, 50]. As for OA treatment, it has been reported that MSCs disappeared from the target tissue quickly after transplantation, but were still able to deliver chondroprotective and immunomodulatory effects [35, 51]. The therapeutic efficacy of stem cell transplantation seems to be independent of their differential ability but mainly depend on the modulation of the microenvironment and stimulation of endogenous regeneration mediated by its paracrine function. In our study, with engineered to secrete the IGF-1 in a “pulse-like” manner, the paracrine function of ADSCs seems magnified. We compared the therapeutic effect of native ADSCs and IGF-1-ADSCs in murine OA model induced by destabilization of medial meniscus (DMM). Our result showed that the injection of ADSCs led to a limited effect on OA pathology in DMM model which was consistent with previous study [52], while IGF-1-ADSCs resulted in effectively ameliorating the progression of OA (Figs. 5, 6). We speculated this might be due to the sufficient secretion of the IGF-1 initiated intrinsic physiological stimulation that continued to drive chondroprotective effect and facilitated the regeneration process. Considering the proposed “hit-and-run” concept, the highly efficient, transient protein expression by modRNA might be beneficial for modulation of the microenvironment and promotion of ECM synthesis in OA.

Currently, a major hurdle of stem cell therapy is the low cell survival rate after transplantation [53, 54]. The majority of transplanted cells die off in the short term, which may result from the hostile microenvironment in OA suffered from hypoxia, insufficiency of metabolic substrates and inflammation. We found that the injected ADSCs could be tracked after 1 week by DiI staining, while signal was only detectable in IGF-1-ADSCs groups after 4 weeks (Fig. 4). We speculated that the overexpression of the IGF-1 enhanced the cell survival. In lined with our findings, it was previously reported that IGF-1 over-expressed MSCs exhibited more resistant to apoptosis and higher cell viability under hypoxia in myocardial infarction model [55, 56]. The mechanism that IGF-1 modRNA transfection increased the survival rate of ADSCs in such OA microenvironment is also of interest and worth being studied in future studies.

Novel delivery systems such as microparticles and hydrogels were developed for the delivery of mRNA and therapeutic protein, which effectively decreased the degradation rates of mRNA/protein and improved transfection/delivery efficiency [57–59]. However, the current delivery systems are difficult to achieve clinical translation due to the biodegradability of the materials and unexpected adverse inflammatory/immunological reactions [60, 61]. Yu et al. have demonstrated that using ADSCs [22] or fibroblasts [18] as cell-carriers for modRNA delivery, can effectively prevent the degradation of modRNA transcripts and highly express target protein without triggering any additional adverse side effects. Compared to delivery system involved foreign body implantation, this autologous cell therapy treatment regime seems to be safer and has more translational potential. Besides, the IGF-1 modRNA transfected ADSCs were not merely carriers of the therapeutic gene but a novel strategy that combined stem-cell therapy with therapeutic protein delivery.

Nevertheless, there are some limitations in this study that need to be addressed. Firstly, a major advantage of a modRNA approach is the ability to simultaneously engineer cells with multiple factors. The combination of other anti-inflammatory or trophic factors such as bone morphogenetic protein 2 (BMP-2) or interleukin 4 (IL-4) might further improve the therapeutic efficiency of stem cell therapy. Secondly, the mechanisms of IGF-1-ADSCs on OA treatment still need further investigations. Previous studies had highlighted that the exosomes from gene-modified stem cells showed different composition of specific miRNA and protein which significantly improved the therapeutic effect [62]. Whether or not MSCs engineered with modRNA could enhance the therapeutic efficacy through exosomes excretion would be an interesting topic for the following studies.

## Conclusion

In this study, we have applied modRNA transfection to generate engineered ADSCs with the ability to secret IGF-1. The application of IGF-1-ADSCs effectively delayed the progression of OA demonstrated by the amelioration of cartilage destruction and the increased expression of anabolic markers of chondrocytes. modRNA transfection rises as a promising platform to enhance stem cell-based therapeutic strategy, which might be an effective approach for OA therapy.

## Supplementary Information

**Additional file 1.** The sequence of IGF-1-modRNA.

## Data Availability

Not applicable.

## Abbreviations

ADSCs: Adipose-derived stem cells
modRNA: Modified mRNA
IGF-1: Insulin-like growth factor 1
OA: Osteoarthritis
ECM: Extracellular matrix
MSCs: Mesenchymal stromal/stem cells
OARSI: The Osteoarthritis Research Society International
SO staining: Safranin Orange staining
GFP: Green fluorescent protein
IL-1β: Interleukin 1 beta
UC-MSCs: Umbilical cord-derived stem cells
BMSCs: Bone marrow mesenchymal stem cells
IL-4: Interleukin 4
BMP-2: Bone morphogenetic protein 2
BMPs: Bone morphogenetic proteins
