# Supplementary material for: Engineered adipose-derived stem cells with IGF-1-modified mRNA ameliorates osteoarthritis development
Source: Stem Cell Res Ther. 2022 Jan 15;13:19. doi: 10.1186/s13287-021-02695-x (PMC8760691; doi:10.1186/s13287-021-02695-x)
Supplement: Supplementary file 1 — Additional file 1. The sequence of IGF-1-modRNA. [file 13287_2021_2695_MOESM1_ESM.docx]

**Supplementary data:**

Table S1 Sequence of IGF-1-modRNA

|  | Sequence |
| --- | --- |
| Mouse IGF-1 | ATGGGGAAAATCAGCAGCCTTCCAACTCAATTATTTAAGATCTGCC  TCTGTGACTTCTTGAAGATAAAGATACACATCATGTCGTCTTCACA  CCTCTTCTACCTGGCGCTCTGCTTGCTCACCTTCACCAGCTCCACC  ACAGCTGGACCAGAGACCCTTTGCGGGGCTGAGCTGGTGGATGCT  CTTCAGTTCGTGTGTGGACCGAGGGGCTTTTACTTCAACAAGCCCA |
